# Identification of High-Risk of Recurrence in Clinical Stage I Non-Small Cell Lung Cancer

**DOI:** 10.3389/fonc.2021.622742

**Published:** 2021-06-07

**Authors:** Yasuhiro Tsutani, Yoshihisa Shimada, Hiroyuki Ito, Yoshihiro Miyata, Norihiko Ikeda, Haruhiko Nakayama, Morihito Okada

**Affiliations:** ^1^ Department of Surgical Oncology, Hiroshima University, Hiroshima, Japan; ^2^ Department of Thoracic Surgery, Tokyo Medical University, Tokyo, Japan; ^3^ Department of Thoracic Surgery, Kanagawa Cancer Center, Yokohama, Japan

**Keywords:** ground-glass opacity, high-resolution computed tomography, recurrence, non-small cell lung cancer, solid component size

## Abstract

**Objective:**

This study aimed to identify patients at a high risk of recurrence using preoperative high-resolution computed tomography (HRCT) in clinical stage I non-small cell lung cancer (NSCLC).

**Methods:**

A total of 567 patients who underwent screening and 1,216 who underwent external validation for clinical stage I NSCLC underwent lobectomy or segmentectomy. Staging was used on the basis of the 8^th^ edition of the tumor–node–metastasis classification. Recurrence-free survival (RFS) was estimated using the Kaplan–Meier method, and the multivariable Cox proportional hazards model was used to identify independent prognostic factors for RFS.

**Results:**

A multivariable Cox analysis identified solid component size (hazard ratio [HR], 1.66; 95% confidence interval [CI] 1.30–2.12; P < 0.001) and pure solid type (HR, 1.82; 95% CI 1.11–2.96; P = 0.017) on HRCT findings as independent prognostic factors for RFS. When patients were divided into high-risk (n = 331; solid component size of >2 cm or pure solid type) and low-risk (n = 236; solid component size of ≤2 cm and part solid type) groups, there was a significant difference in RFS (HR, 5.33; 95% CI 3.09–9.19; 5-year RFS, 69.8% *vs.* 92.9%, respectively; P < 0.001). This was confirmed in the validation set (HR, 5.32; 95% CI 3.61–7.85; 5-year RFS, 72.0% *vs.* 94.8%, respectively; P < 0.001).

**Conclusions:**

In clinical stage I NSCLC, patients with a solid component size of >2 cm or pure solid type on HRCT were at a high risk of recurrence.

## Introduction

Early-stage non-small cell lung cancer (NSCLC) is frequently detected using more complex procedure, such as high-resolution computed tomography (HRCT) and widespread use of low-dose helical computed tomography (CT) for tumor screening (1, 2). The occasion to treat patients with early-stage NSCLC has increased, and pulmonary resection plays a main role in such treatment. Complete resection would be expected to lead a good prognosis for stage I NSCLC. However, complete resection does not always ensure the cure of disease and the 5-year disease-free survival rate for stage IA NSCLC is 84.3% and for stage IB NSCLC is 65.8% (3).

Although the tumor–node–metastasis (TNM) classification can stratify patients into different prognostic groups, stage I NSCLC is considered to be a heterogeneous group. Therefore, useful parameters are needed to predict postoperative recurrence when deciding on treatment strategies, such as the application of sublobar resection and additional perioperative systemic therapy. A high maximum standardized uptake value (SUVmax) with [18F]-fluoro-2-deoxy-D-glucose (FDG) positron emission tomography (PET)/CT reflects tumor invasiveness and has a negative impact on prognosis in patients with resected early-stage NSCLC (2, 4). However, because imaging protocols, equipment, and measurement methods vary between institutions, the optimal SUVmax cutoff value also differs by institution and between studies. Therefore, using FDG-PET/CT as a prognostic factor in a universal setting is challenging at present.

One of the important proposals in the 8^th^ edition of the T classification of lung cancer is the size of the solid component excluding ground-glass opacity (GGO) to classify clinical T factors (5, 6). In the present study, we aimed to identify patients at a high risk of postoperative recurrence in clinical stage I NSCLC using preoperative HRCT, which is a globally applicable method. High-risk patients may be candidates for neoadjuvant therapy even in clinical stage I NSCLC. The primary endpoint of this study was recurrence-free survival (RFS).

## Materials and Methods

### Patients

**Figure 1:**
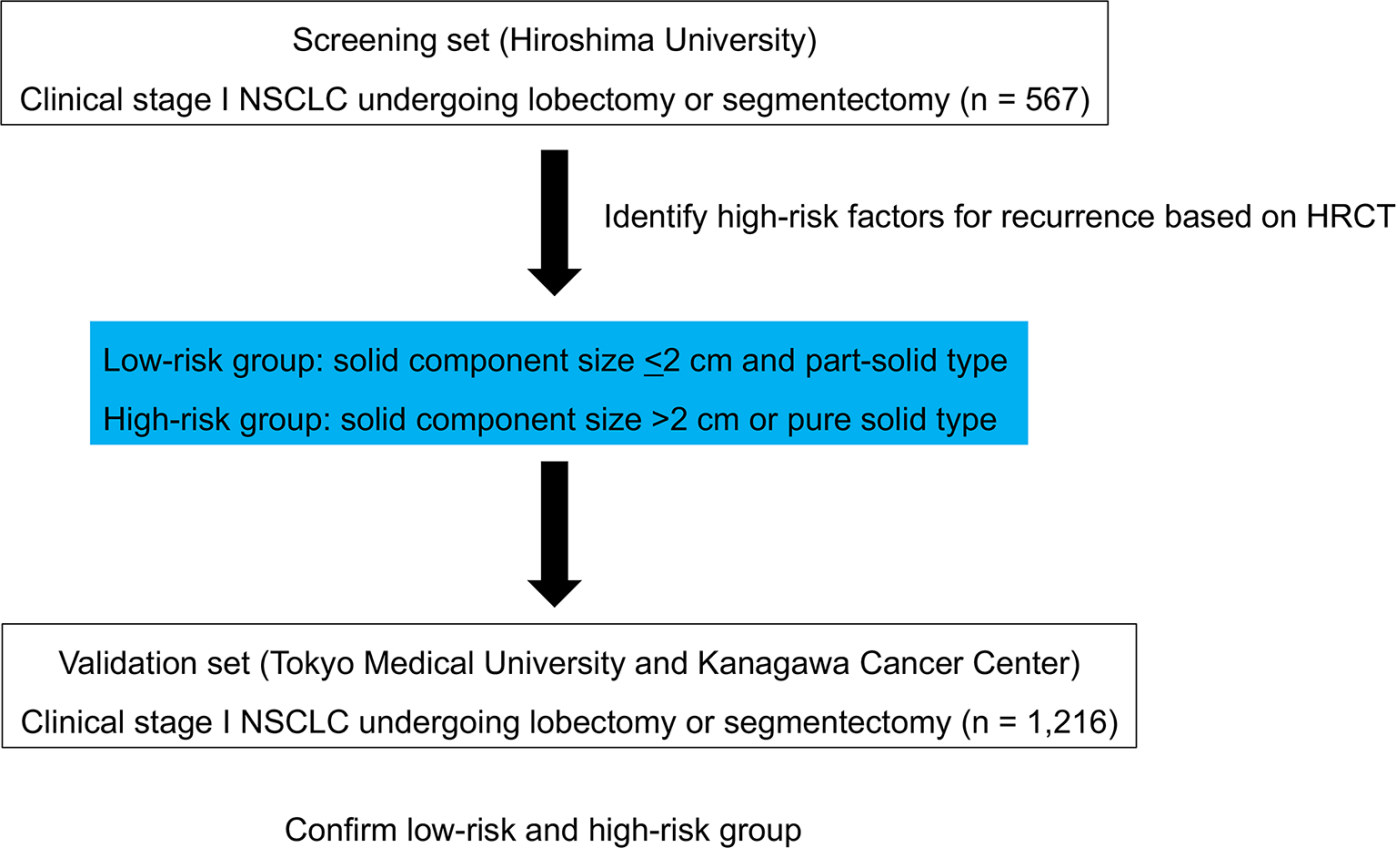
Scheme of the study.

As the screening set, a total of 567 consecutive patients with clinical stage I NSCLC who underwent lobectomy or segmentectomy with systematic lymph node dissection at Hiroshima University between January 1^st^ 2010 and December 31^st^ 2016 were enrolled. All patients were staged according to the TNM Classification of Malignant Tumors, 8^th^ edition (7). Endobronchial ultrasonography and mediastinoscopy were not routinely performed. Lymph node metastasis was determined as negative when swollen mediastinal or hilar lymph nodes measuring short axis of > 1 cm were not evident on HRCT, and FDG did not accumulate an SUXmax of >1.5 in these lymph nodes according to FDG-PET. The inclusion criteria included preoperative staging determined by HRCT and FDG-PET/CT and curative surgery with lobectomy or segmentectomy without induction therapy. The screening set of patients formed the high-risk group for recurrence. To externally validate the high-risk group, we used a combined cohort of two independent sets of 1,216 consecutive patients who underwent lobectomy or segmentectomy with systematic lymph node dissection (Tokyo Medical University and Kanagawa Cancer Center) between January 1^st^, 2010, and December 31^st^, 2016. The inclusion criteria were the same as those for the screening set (**Figure 1**).

The institutional review boards of the participating institutions approved this retrospective review, which was based on a prospective database, and waived the requirement for informed consent from individual patients (Kanagawa Cancer Center: February 28th, 2013; 24-KEN-54; Tokyo Medical University Hospital: February 25th, 2015; SH2969; Hiroshima University Hospital: June 13th, 2018; E-1216).

### HRCT

Sixteen-row multidetector CT was used to obtain chest images independent of subsequent FDG-PET/CT examinations. For high-resolution tumor images, the following parameters were used: 120 kVp, 200 mA, 1–2-mm section thickness, 512 × 512-pixel resolution, 0.5–1.0-s scan time, a high spatial reconstruction algorithm with a 20-cm field of view, and mediastinal (level, 40 HU; width, 400 HU) and lung (level, −600 HU; width, 1600 HU) window settings. GGO was defined as a misty increase in lung attenuation without obscuring the underlying vascular markings. The size of the solid component of the tumor was defined as the maximum dimension of the solid component measured using lung window settings, excluding GGO (8). Pure solid tumor was defined as a tumor without GGO component. Part-solid tumor was defined as a tumor with GGO component. CT scans were reviewed and tumor sizes were determined by radiologists from each institution.

### Pathological Examination

Tumor size was defined as the maximum dimension of the invasive tumor component, excluding the lepidic growth component described previously (9). Lymphatic and vascular invasion were assessed by immunohistochemistry with D2-40, which stains lymphatic ducts, and elastic Van Gieson staining of vessel elastic fibers. Lymphatic and vascular invasion were positive when penetration was detected as an extension of the malignant neoplasm. To evaluate pleural invasion, elastic tissue fibers were subject to elastic Van Gieson staining. Pleural invasion was defined as positive if cancer had invaded beyond the elastic layer, including invasion into the visceral pleural surface or neighboring organs. Histological examinations were determined by pathologists at each institution.

### Follow-Up Evaluation

All patients who underwent lung resection were followed up from the day of surgery. Postoperative follow-up procedures, including a physical examination, chest roentgenogram every three months, and chest and abdominal CT examinations every six months, were performed for the first two years. Subsequently, a physical examination and chest roentgenogram were performed every six months, and a chest CT examination was performed every year.

### Statistical Analysis

Data is presented as number (%) or median and interquartile range (IQR) unless otherwise stated. The χ (2) test was used to compare the frequencies of categorical variables. Receiver operating characteristic curve of the solid component size for the prediction of recurrence were generated to determine the cutoff value that yielded optimal sensitivity and specificity. An independent-samples t-test was used to compare continuous variables. RFS was defined as the interval from the day of surgery until the first event (relapse or death from any cause) or right censoring on the day of final follow up. Overall survival (OS) was defined as the time from the day of surgery until death from any cause or right censoring at the day of final follow up. The Kaplan–Meier method was used to analyze RFS and OS. The log-rank test was used to assess differences in RFS and OS between groups. The multivariable Cox proportional hazards model was used to identify independent prognostic factors for RFS. The multivariable logistic regression analysis was used to identify independent predictive factors for lymph node metastasis. A P value of <0.05 was considered statistically significant. JMP 14.0 (SAS Institute, Cary, NC, USA) was used to statistically analyze the data.

## Results

### Screening Set

Patient characteristics from the screening set (n = 567) are shown in **Table 1**.

**Table 1:** Patient characteristics in the screening set and validation set.

		Screening set (n = 567)	Validation set (n = 1,216)	P value
Age, median (IQR)		68 (62-74)	69 (63-75)	0.090
Gender	Male	334 (58.9%)	603 (49.6%)	<0.001
Smoking history		321 (56.6%)	657 (54.0%)	0.307
Solid component size (cm), median (IQR)		1.6 (1.1-2.5)	1.7 (1.1-2.4)	0.828
Pure solid type		276 (48.7%)	502 (41.3%)	0.003
Histology	Adenocarcinoma	452 (79.7%)	1,033 (85.0%)	0.049
	Squamous cell carcinoma	67 (11.8%)	109 (9.0%)	
	Others	48 (8.5%)	73 (6.0%)	
Procedure	Lobectomy	366 (64.6%)	1,060 (87.2%)	<0.001
	Segmentectomy	201 (35.4%)	156 (12.8%)	
Lymphatic invasion		111 (19.6%)	265 (21.8%)	0.283
Vascular invasion		140 (24.7%)	353 (29.0%)	0.055
Pleural invasion		87 (15.3%)	230 (18.9%)	0.064
Lymph node metastasis		47 (8.3%)	138 (11.3%)	0.045
Pathological stage	0	20 (3.5%)	43 (3.5%)	0.002
	IA1	118 (20.8%)	350 (28.8%)	
	IA2	171 (30.2%)	321 (26.4%)	
	IA3	83 (14.6%)	130 (10.7%)	
	IB	93 (16.4%)	179 (14.7%)	
	IIA	7 (1.2%)	16 (1.3%)	
	IIB	51 (9.0%)	110 (9.1%)	
	IIIA	24 (4.2%)	58 (4.8%)	
	IIIB	0 (0%)	9 (0.7%)	
Adjuvant chemotherapy	Yes	189 (33.3%)	240	<0.001

IQR, interquartile range.

A multivariable analysis revealed that age (hazard ratio [HR], 1.03; P = 0.002), solid component size (HR, 1.66; P <0.001), and pure solid type (HR, 1.82; P = 0.017) were independent prognostic factors for RFS (**Table 2**). The optimal cutoff value of solid component size to predict recurrence was set as 2.0 cm from the receiver operating characteristic curve (**Supplementary Figure 1**).

**Table 2:** Multivariable analysis of recurrence-free survival.

Screening set		Univariable analysis			Multivariable analysis		
Variable		HR	95% CI	P value	HR	95% CI	P value
Age		1.04	1.02-1.07	<0.001	1.03	1.01-1.05	0.002
Gender	Male (*vs.* female)	1.87	1.24-2.83	0.003	1.07	0.63-1.83	0.792
Smoking history		2.15	0.42-3.26	0.001	1.48	0.84-2.61	0.173
Solid component size (cm)		2.01	1.66-2.46	<0.001	1.66	1.30-2.12	<0.001
Pure solid type		3.31	2.17-5.04	<0.001	1.82	1.11-2.96	0.017
Histology	Adenocarcinoma (*vs.* non-adenocarcinoma)	0.39	0.26-0.58	<0.001	0.98	0.61-1.61	0.915
Procedure	Lobectomy (*vs.* segmentectomy)	1.46	0.95-2.24	0.081	1.02	0.64-1.55	0.944

HR, hazard ratio; CI, confidence interval.

A multivariable analysis revealed that age (odds ratio [OR], 1.03; P = 0.002), gender (male, OR, 1.67; P = 0.013), solid component size (OR, 1.75; P <0.001), pure solid type (OR, 2.85; P<0.001), and lobectomy (OR, 3.25; P = 0.001) were independent predictive factors for lymph node metastasis (**Table 3**).

**Table 3:** Multivariable analysis of lymph node metastasis.

Screening set		Univariable analysis			Multivariable analysis		
Variable		OR	95% CI	P value	OR	95% CI	P value
Age		1.02	1.00-1.03	0.033	1.03	1.01-1.04	0.002
Gender	Male (*vs.* female)	1.67	1.22-2.30	0.001	1.67	1.11-2.50	0.013
Smoking history		1.26	0.93-1.72	0.136	0.67	0.44-1.02	0.064
Solid component size (cm)		2.21	1.97-2.61	<0.001	1.75	1.44-2.13	<0.001
Pure solid type		4.13	2.94-5.82	<0.001	2.85	1.94-4.20	<0.001
Histology	Adenocarcinoma (*vs.* non-adenocarcinoma)	0.61	0.42-0.87	0.009	1.28	0.84-1.95	0.257
Procedure	Lobectomy (*vs.* segmentectomy)	5.44	2.76-10.75	<0.001	3.25	1.61-6.56	0.001

OR, odds ratio; CI, confidence interval.

**Figure 2:**
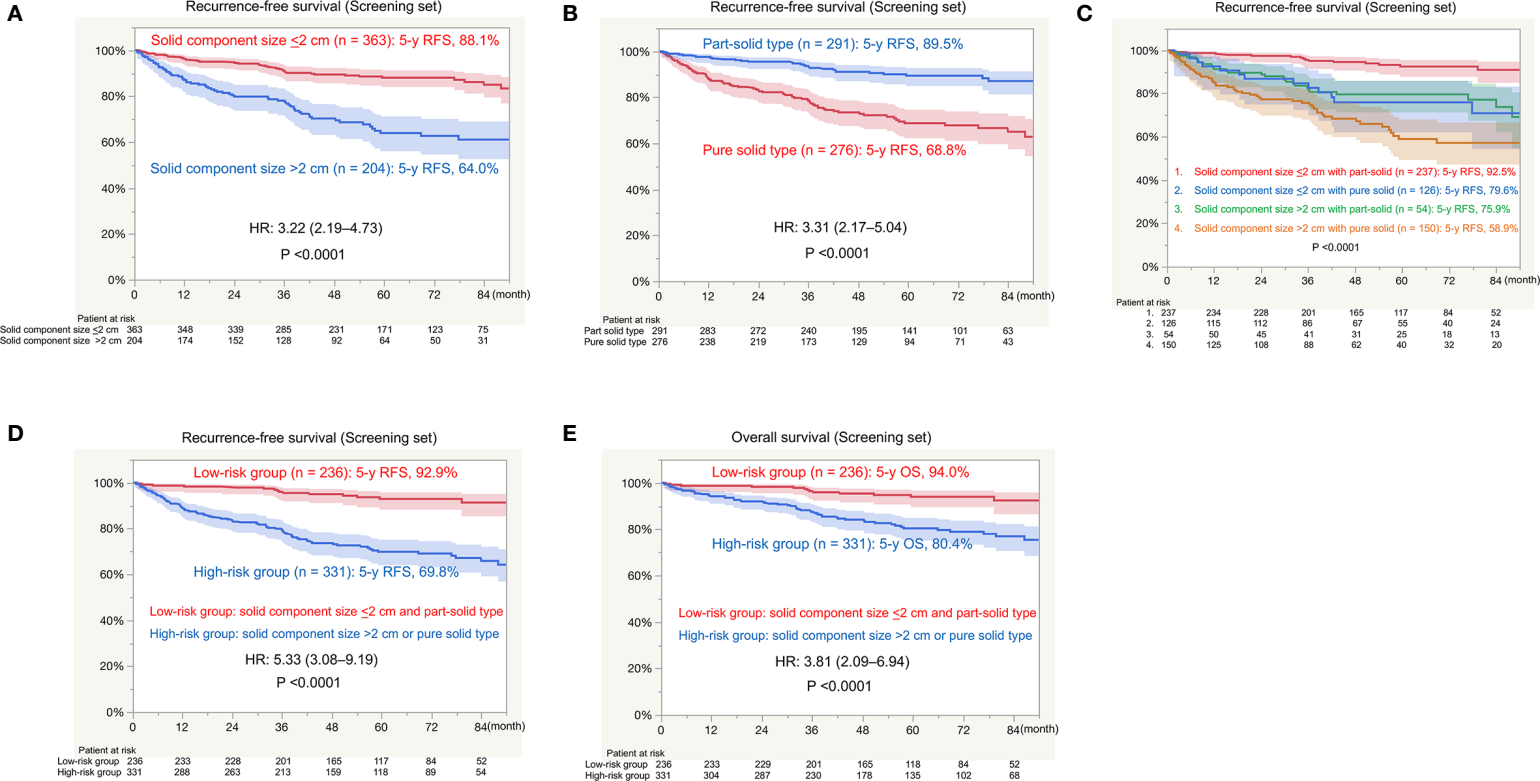
Survival in the screening set. RFS, recurrence-free survival; HR, hazard ratio; OS, overall survival. **(A)** RFS between patients with a solid component size of ≤2 cm and those with a solid component size of >2 cm. **(B)** RFS between patients with part-solid tumors and those with pure solid tumors. **(C)** RFS among patients with a solid component size of ≤2 cm with part-solid tumors or pure solid tumors and those with a solid component size of >2 cm with part-solid tumors or pure solid tumors. **(D)** RFS between the low-risk group (solid component size of ≤2 cm and a part-solid tumor) and high-risk group (solid component size of >2 cm and a pure solid tumor). **(E)** OS between the low-risk group (solid component size of ≤2 cm and a part-solid tumor) and high-risk group (solid component size of >2 cm or a pure solid tumor).

There was a significant difference in RFS between patients with a solid component size of ≤2 cm (n = 363; 5-year RFS, 88.1%) and those with a solid component size of >2 cm (n = 204; 5-year RFS, 64.0%; P < 0.0001; **Figure 2A**). There was a significant difference in RFS between patients with part-solid tumors (n = 291; 5-year RFS, 89.5%) and those with pure solid tumors (n = 276; 5-year RFS, 68.8%; P < 0.0001; **Figure 2B**). When patients were divided into four groups based on the solid component size and pure or part-solid type, RFS of patients with a solid component size of ≤2 cm with a part-solid tumor (n = 129) were favorable with a 5-year RFS of 92.5%. RFS in the other three groups was similar; 5-year RFS was 79.6% for patients with a solid component size of ≤2 cm with a pure solid tumor (n = 126), 75.9% for those with a solid component size of >2 cm with a part solid tumor (n = 54), and 58.9% for those with a solid component size of >2 cm with a pure solid tumor (n = 150; **Figure 2C**). Based on these findings, we defined patients as at a low risk of recurrence with a solid component size of <2 cm and part-solid type, and at a high risk of recurrence with a solid component size of >2 cm or pure solid type. Pathological findings, such as histology; invasive component size; lymphatic, vascular and pleural invasion; and lymph node metastasis were significantly different between the low-risk and high-risk groups (**Table 4**). There was a significant difference in RFS between the low-risk (n = 236; 5-year RFS, 92.9%) and high-risk (n = 331; 5-year RFS, 69.8%; HR, 5.33; P < 0.0001; **Figure 2D**) groups. There was a significant difference in OS between the low-risk group (n = 236; 5-year OS, 94.0%) and the high-risk group (n = 331; 5-year OS, 80.4%; HR, 3.81; P < 0.0001; **Figure 2E**).

**Table 4:** Comparison of pathological findings between low-risk and high-risk groups.

		Screening set			Validation set		
		Low risk (n = 236)	High risk (n = 331)	P value	Low risk (n = 553)	High risk (n = 663)	P value
Histology	Adenocarcinoma	234 (99.2%)	218 (65.9%)	<0.001	539 (97.5%)	494 (74.5%)	<0.001
	Squamous cell carcinoma	1 (0.42%)	66 (19.9%)		11 (2.0%)	98 (14.8%)	
	Others	1 (0.42%)	47 (14.2%)		3 (0.5%)	71 (10.7%)	
Invasive component size (cm)		1.1 (0.6-1.8)	2.0 (1.5-2.9)	<0.001	0.8 (0.3-1.4)	2.0 (1.5-2.8)	<0.001
Lymphatic invasion		13 (5.5%)	98 (29.6%)	<0.001	45 (8.1%)	220 (33.2%)	<0.001
Vascular invasion		13 (5.5%)	127 (38.4%)	<0.001	34 (6.2%)	319 (48.1%)	<0.001
Pleural invasion		12 (5.1%)	75 (22.7%)	<0.001	26 (4.7%)	204 (30.8%)	<0.001
Lymph node metastasis		0 (0%)	47 (14.2%)	<0.001	18 (3.3%)	120 (18.1%)	<0.001
Pathological stage	0	20 (8.5%)	0 (0%)	<0.001	38 (6.9%)	5 (0.8%)	<0.001
	IA1	85 (36.0%)	33 (10.0%)		287 (51.9%)	63 (9.5%)	
	IA2	83 (35.2%)	88 (26.6%)		155 (28.0%)	166 (25.0%)	
	IA3	22 (9.3%)	61 (18.4%)		28 (5.1%)	102 (15.4%)	
	IB	14 (5.9%)	79 (23.9%)		22 (4.0%)	157 (23.7%)	
	IIA	3 (1.3%)	4 (1.2%)		1 (0.2%)	15 (2.3%)	
	IIB	9 (3.8%)	42 (12.7%)		14 (2.5%)	96 (14.5%)	
	IIIA	0 (0%)	24 (7.3%)		7 (1.3%)	51 (7.7%)	
	IIIB	0 (0%)	0 (0%)		1 (0.2%)	8 (1.2%)	
Adjuvant chemotherapy	Yes	57 (24.2%)	132 (39.9%)	<0.001	48 (8.7%)	192 (29.0%)	<0.001

IQR, interquartile range.

### Validation Set

The characteristics of patients in the validation set (n = 1,216) are shown in **Table 1**.

When patients were divided into the low risk of recurrence group (n = 553) and the high risk of recurrence group (n = 663), there were significant differences in pathological findings, such as histology; invasive component size; lymphatic, vascular, and pleural invasion; and lymph node metastasis between the two groups (**Table 4**).

**Figure 3:**
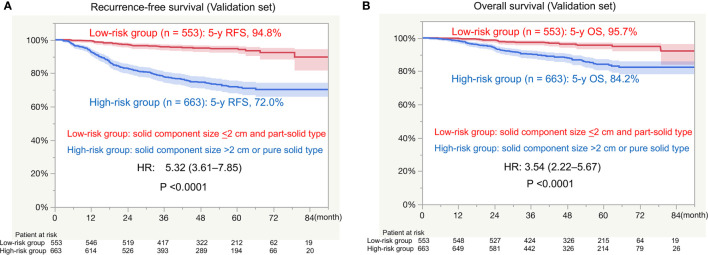
Survival in the external validation set. RFS, recurrence-free survival; HR, hazard ratio; OS, overall survival. **(A)** RFS between the low-risk group (solid component size of ≤2 cm and a part-solid tumor) and high-risk group (solid component size of >2 cm and a pure solid tumor). **(B)** OS between the low-risk group (solid component size of ≤2 cm and a part-solid tumor) and high-risk group (solid component size of >2 cm and a pure solid tumor).

There was a significant difference in RFS between the low-risk group (5-year RFS, 94.8%) and high-risk group (5-year RFS, 72.0%; HR, 5.32; P < 0.0001; **Figure 3A**). There was a significant difference in OS between the low-risk group (5-year OS, 95.7%) and high-risk group (5-year OS, 84.2; HR, 3.54; P < 0.0001; **Figure 3B**).

## Discussion

We identified patients at a high risk of recurrence using preoperative HRCT for clinical stage I NSCLC. Patients with a solid component size of >2 cm or a pure solid tumor were at a high risk of recurrence. The HR values of RFS and OS in the low-risk group in the screening set were 5.33 and 3.81, respectively. These findings were confirmed in the external validation set with high concordance, with a HR of 5.32 for RFS and 3.54 for OS.

Several studies have reported the utility of solid component size to predict pathological tumor invasiveness and prognosis compared with whole tumor size (8, 10–12). As recommended in the 8^th^ edition of the T classification (6), using solid component size as a prognostic factor seems to be reasonable from the results of the present study, which show that solid component size is an independent prognostic factor for RFS.

The presence of GGO was also reported as a prognostic factor in previous studies (13–15). The multivariable analysis of pure solid versus part-solid tumors showed this characteristic as an independent unfavorable prognostic factor for RFS, which is consistent with previous reports. Although survival after surgical resection for tumors with GGO was usually favorable (13–15), we need to pay attention to GGO tumors with a large solid component size. As shown in **Figure 2C**, RFS in patients with a part-solid tumor with a solid component size of >2 cm is similar to that of pure solid tumors in this study. This finding was also supported by a previous study (16).

The group at a high risk of recurrence reflected the pathological findings in the current study. The frequency of non-adenocarcinoma histology; a larger invasive component size; lymphatic, vascular, and pleural invasion; and lymph node metastasis were significantly higher in the high-risk group compared with the low-risk group in both the screening and validation sets. In the multivariable analysis, solid component size and the pure solid type were proven to be predictive factors for lymph node metastasis. Patients at a high-risk of recurrence were also at high-risk of lymph node metastasis.

Preoperative prediction of the risk of postoperative recurrence is useful to decide treatment strategies. The excellent prognosis would be expected after lobectomy or segmentectomy with lymph node dissection in patients at a low risk of recurrence that show minimal malignant pathological findings in our study. There seems to be no role of neoadjuvant therapy in low-risk patients. However, when nodal metastasis is proven, adjuvant chemotherapy should be considered. In contrast, patients at a high risk of recurrence should undergo standard therapy, such as lobectomy with systematic lymph node dissection, because they potentially have lymph node metastasis with a probability of approximately 20%. Although the use of perioperative adjuvant therapy for stage I NSCLC has not been established, additional systemic therapy, such as neoadjuvant chemotherapy or immunotherapy may be needed to improve outcomes in high-risk patients with stage I NSCLC. We are currently conducting a multicenter pilot study of neoadjuvant anti-programmed cell death-1 antibody for high-risk clinical stage I NSCLC with a solid component size of >2 cm or pure solid type on HRCT (17).

The current study has some limitations. Only HRCT was used to predict risk factors for postoperative recurrence while several previous studies suggested that SUVmax on FDG-PET/CT was a promising predictor of prognosis (2, 4, 10). Although no standardization of SUVmax on FDG-PET/CT in a global setting can be used at present, further studies should be done to elucidate the significance of FDG-PET using a novel standardization method, such as semiquantitative PET evaluation (18). Also, we did not routinely perform invasive mediastinal staging for node-negative tumors, although invasive staging is recommended for tumors >3 cm (mainly adenocarcinoma with high FDG uptake) in the guideline (19). High-risk patients may also be candidates of invasive staging. Although we identified high-risk patients using preoperative HRCT findings to decide the application of neoadjuvant therapy, postoperative adjuvant therapy should be considered on the basis of pathological findings such as nodal involvement and pleural invasion.

In conclusion, we established patients at a high risk of recurrence using preoperative HRCT for clinical stage I NSCLC with high concordance HR in the external validation set. Patients with a solid component size of >2 cm or pure solid tumors are potential candidates for perioperative systemic therapy to prevent postoperative recurrence.

## Data Availability Statement

The raw data supporting the conclusions of this article will be made available by the authors, without undue reservation.

## Ethics Statement

The studies involving human participants were reviewed and approved by Hiroshima University IRB, Kanagawa Cancer Center IRB, and Tokyo Medical University Hospital IRB. Written informed consent for participation was not required for this study in accordance with the national legislation and the institutional requirements.

## Author Contributions

YT: conceptualization, data curation, methodology, data analysis, and writing-original draft. YS: data collections, writing-review, and editing. HI: data collections, writing-review, and editing. YM: writing-review and editing. NI: writing-review and editing. HN: writing-review and editing. MO: writing-review and editing, supervision. All authors contributed to the article and approved the submitted version.

## Conflict of Interest

The authors declare that the research was conducted in the absence of any commercial or financial relationships that could be construed as a potential conflict of interest.

## Supplementary Material

Supplementary Figure 1 Receiver operating characteristic curve of solid component size to predict recurrence. AUC, area under the curve.
