# Supplementary figures and images for: Identification of High-Risk of Recurrence in Clinical Stage I Non-Small Cell Lung Cancer
Source: Front Oncol. 2021 Jun 7;11:622742. doi: 10.3389/fonc.2021.622742 (PMC8215653; doi:10.3389/fonc.2021.622742)

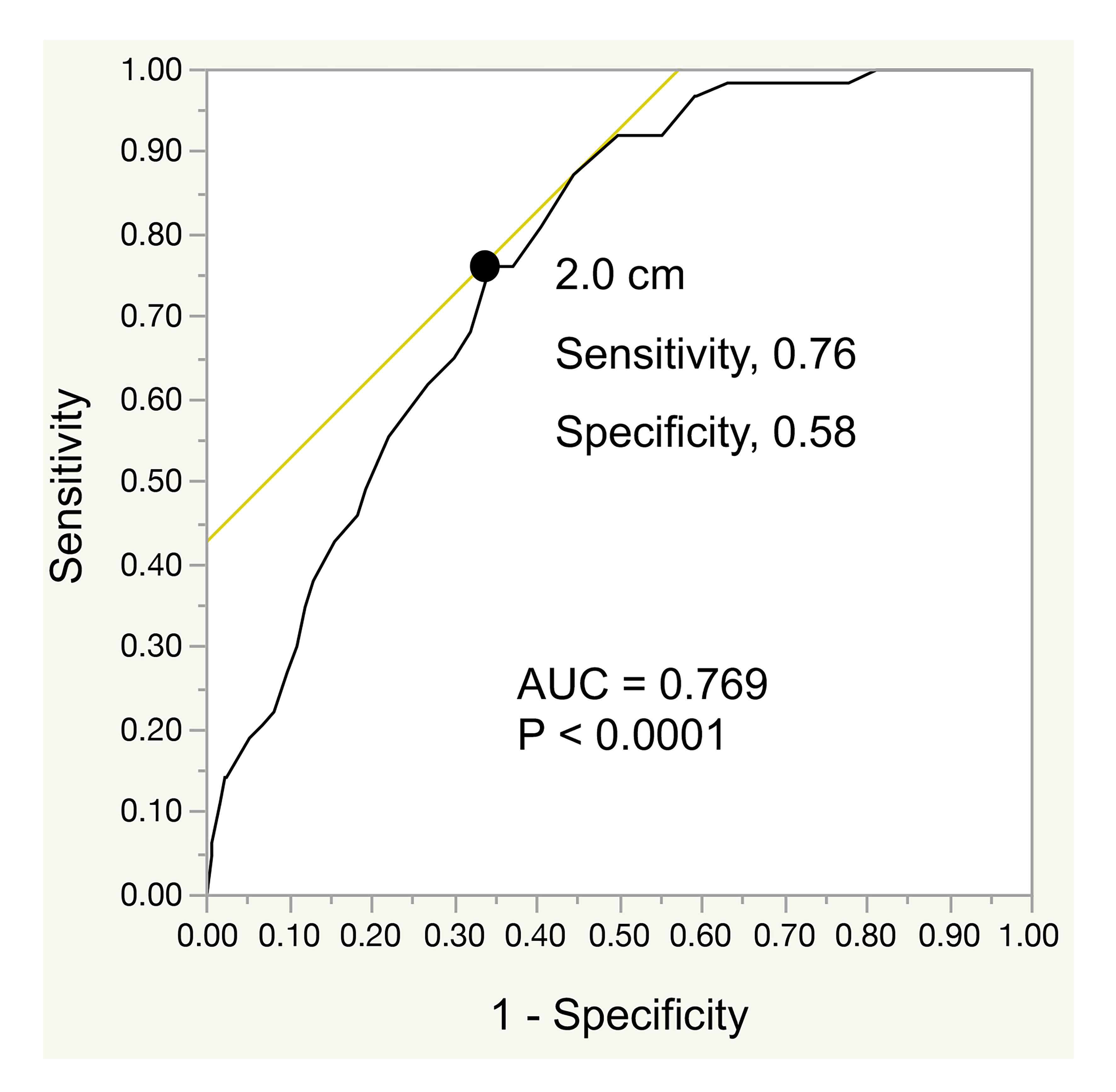

Supplement: Supplementary Figure 1 — Receiver operating characteristic curve of solid component size to predict recurrence. AUC, area under the curve. [file Image_1.tif]
